# Current Status and Future Direction of Antithrombotic Therapy for Patients with STEMI Undergoing Primary PCI

**DOI:** 10.31083/j.rcm2309297

**Published:** 2022-09-05

**Authors:** Rosanne F. Vogel, Ronak Delewi, Lina Badimon, Dominick J. Angiolillo, Georgios J. Vlachojannis

**Affiliations:** ^1^Department of Cardiology, University Medical Center Utrecht, Utrecht University, 3584CX Utrecht, The Netherlands; ^2^Department of Cardiology, Amsterdam UMC location AMC, University of Amsterdam, 1105AZ Amsterdam, The Netherlands; ^3^Cardiovascular Science Program (ICCC), IR-Hospital de la Santa Creu I Santa Pau-IIBSantPau, CiberCV, Autonomous University of Barcelona, 08025 Barcelona, Spain; ^4^Department of Cardiology, University of Florida College of Medicine, Jacksonville, FL 32209, USA

**Keywords:** ST-segment elevation myocardial infarction, STEMI, antithrombotic therapy, antiplatelet therapy, anticoagulation, primary percutaneous coronary intervention

## Abstract

Since the introduction of the first pharmacological therapy for the treatment of patients with acute myocardial infarction in the early 20th century, treatment of myocardial infarction has evolved extensively throughout the years. Mechanical revascularization therapies such as the percutaneous transluminal coronary angioplasty, combined with the ongoing development of pharmacological therapies have successfully improved the survival of patients with acute myocardial infarction. To date, antiplatelet therapy (consisting of aspirin and an oral ${\mathrm{P2Y}}_{12}$ inhibitor) and anticoagulation therapy represent the main stay of pharmacological treatment in patients with ST-segment elevation myocardial infarction (STEMI) undergoing percutaneous coronary intervention (PCI). The routine use of clopidogrel as antiplatelet agent has been largely replaced by the use of the more potent ${\mathrm{P2Y}}_{12}$ inhibitors ticagrelor and prasugrel. Unfractionated heparin remains the preferred anticoagulant therapy, despite the development of other anticoagulants, including enoxaparin and bivalirudin. To date, limited evidence exists supporting a pre-hospital initiation of antiplatelet and anticoagulant therapy in STEMI patients. The use of potent intravenous antiplatelet agents, including the glycoprotein IIb/IIIa inhibitors and the intravenous ${\mathrm{P2Y}}_{12}$ inhibitor cangrelor, is currently restricted to specific clinical settings. While several potent antithrombotic agents already exist, the search for novel potent antithrombotic agents continues, with a focus on balancing antithrombotic properties with an improved safety profile to reduce excess bleeding. This review provides an overview of currently available pharmacological therapies for the treatment of STEMI patients undergoing primary PCI, and an outlook for the ongoing development of novel agents in this field.

## 1. Introduction

**Fig. 1.**
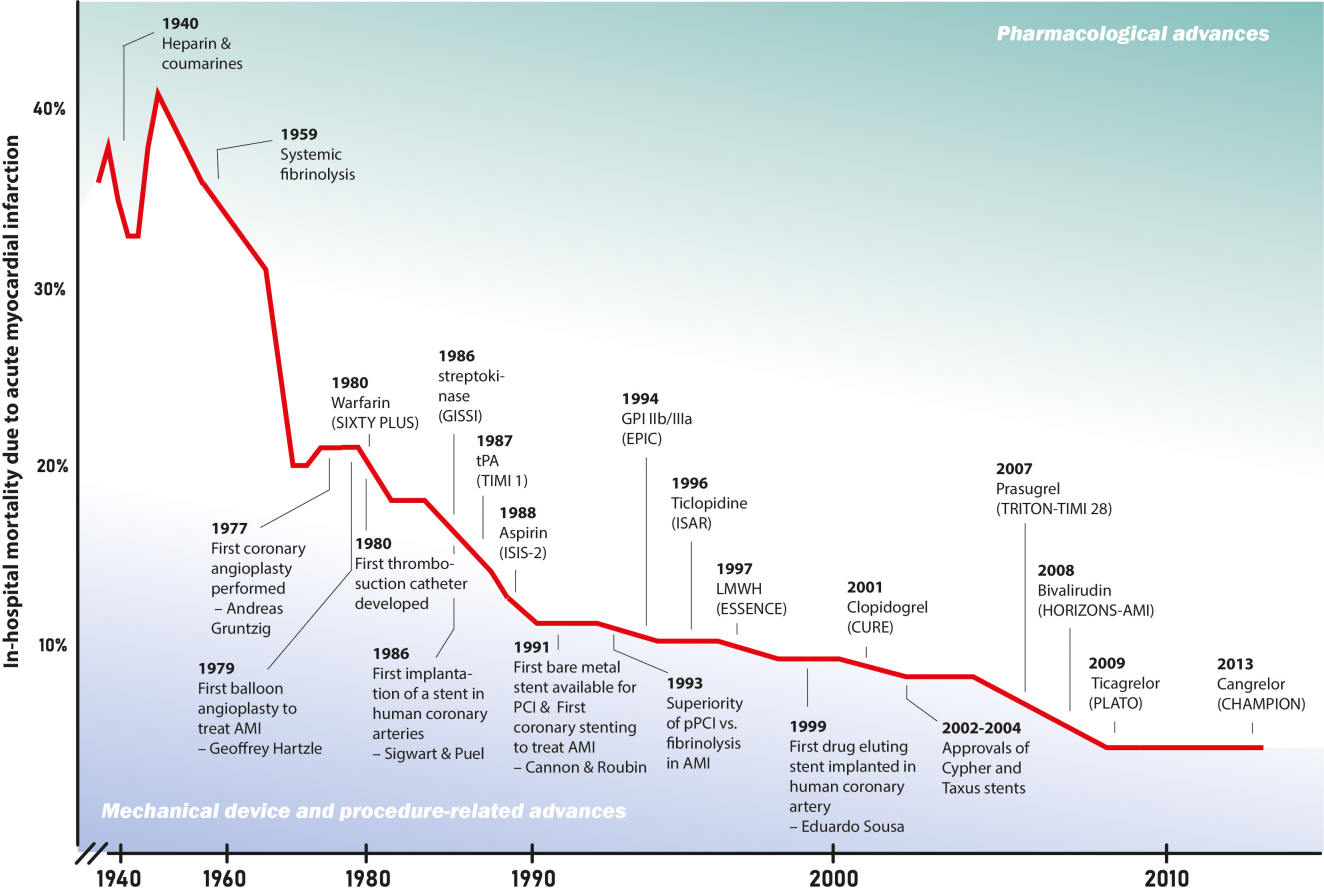
**Decline in in-hospital mortalitiy after acute myocardial infarction in relation to scientific advances**. The timeline desplays the decline of in-hospital mortality after acute myocardial infarction over the last century, along with major advances in reperfusion therapy including inovations in pharmacotherapy, and in procedure and medical devices [1, 2, 3, 4, 5, 6]. CHAMPION, Cangrelor Versus Standard Therapy to Achieve Optimal Management of Platelet Inhibition; CURE, Clopidogrel in Unstable Angina to Prevent Recurrent Events; ESSENCE trial, Efficacy and Safety of Subcutaneous Enoxaparin versus intravenous unfractionated heparin in non–Q-wave Coronary Events; EPIC, Evaluation of 7E3 for the Prevention of Ischemic Complications Study; GISSI, Italian Group for the Study of Streptokinase in Myocardial Infarction; GPI, glycoprotein inhibitor; HORIZONS-AMI, Harmonizing Outcomes With Revascularization and Stents in Acute Myocardial Infarction; ISAR, The Intracoronary Stenting and Antithrombotic Regimen trial; ISIS-2, Second International Study of Infarct Survival; LMWH, low molecular weight heparin; PCI, percutaneous coronary intervention; PLATO, Platelet Inhibition and Patient Outcomes; SIXTY PLUS, Sixty Plus Reinfarction Study; TIMI 1, Thrombolysis in Myocardial Infarction 1; TRITON-TIMI 38, Trial to Assess Improvement in Therapeutic Outcomes by Optimizing Platelet Inhibition with Prasugrel–Thrombolysis in Myocardial Infarction.

Antithrombotic therapy represents an indispensable component of the management of patients with ST-segment elevation myocardial infarction (STEMI) undergoing primary percutaneous coronary intervention (PCI). The advancements of antithrombotic therapies—which have evolved along with developments in interventional devices and refinements of procedural techniques—has contributed to the steep decline in mortality and morbidity in patients with STEMI (Fig. 1, Ref. [1, 2, 3, 4, 5, 6]). Optimizing the use of existing antithrombotic therapies by clarifying the current gaps in evidence and developing new agents with improved safety and efficacy profiles, are highly pursued objectives (Table 1, Ref. [7]). The present review provides an overview of past and current antithrombotic therapies in patients with STEMI who are undergoing primary PCI and examines future developments.

**Table 1.** **A selected overview of gaps in evidence concerning currently available antithrombotic agents in STEMI patients undergoing primary PCI**.

What is the best antithrombotic regimen in patients who have an indication for oral anticoagulants?
What is the optimal timing for the loading dose of oral ${\mathrm{P2Y}}_{12}$ inhibitors?
What is the optimal oral ${\mathrm{P2Y}}_{12}$ inhibitor in STEMI?
Is there a role of pre-treatment (e.g., in a pre-hospital setting) with i.v. antithrombotic agents (role of i.v. ${\mathrm{P2Y}}_{12}$ inhibition, or newer i.v. or s.c. GPI’s)?
Role of GPI in contemporary primary PCI?
What is the role of potent ${\mathrm{P2Y}}_{12}$ inhibitors in patients undergoing fibrinolysis?
What is the role of aspirin in the era of potent antiplatelet agents?

Aapted from the ESC Guidelines for the management of acute myocardial infarction in patients presenting with ST-segment elevation: The Task Force for the management of acute myocardial infarction in patients presenting with ST-segment elevation of the European Society of Cardiology (ESC) [7].

## 2. Historic Perspective of Antithrombotics

Over the course of 80 years, mechanical revascularization and pharmacological treatment options have evolved extensively for patients with STEMI. While the German physician Friedrich Hoffmann (1660–1742) already suspected that some heart diseases might be related to a “reduced passage of the blood within the coronary arteries”, it was the American pathologist Ludwig Hektoen (1863–1951) who in 1889 recognized that acute myocardial infarction (AMI) was a disease caused by thrombotic coronary occlusions “secondary to sclerotic changes in the coronaries” [8, 9]. At that time, AMI was considered a fatal disease and the only available treatment was prolonged bed rest for weeks.

In 1923, the first symptomatic therapy regimen for patients with AMI was published by Joseph Wearn [10]. It included recommendations on fluid restriction, digitalis use to prevent pulmonary congestion, caffeine use to prevent hypotension and conduction blocks, and absolute bed rest. In addition to the risk of hypotension and arrhythmias, patients surviving the acute phase of AMI were at risk of pulmonary embolisms—a potentially lethal complication—as a result of their immobilization due to prolonged bedrest. In the 1950s, the first antithrombotic agents—unfractionated heparin (UFH) and coumarin derivates—became available for the treatment of AMI [11]. This pharmacologic option had initially emerged from the need to prevent pulmonary embolisms in AMI patients. However it was quickly recognized that anticoagulation therapy additionally decreased the rate of mortality within five weeks by nearly 10% [12]. Nonetheless, knowledge about the pharmacologic mechanism of action or the pathophysiology of thrombosis was still limited at that time.

During the 1970s, some important new pathophysiological insights on the role of platelets in AMI were gained. Researchers discovered that activated platelet levels were markedly increased in patients with AMI and that platelet aggregation played an important role in intraluminal thrombus formation [13]. These findings triggered a profound interest in the development of platelet-targeted therapies in addition to the anticoagulation therapy that already was used regularly at that time [14, 15].

In parallel, the first mechanical coronary revascularization was performed by Andreas Grüntzig in 1977 in a patient with stable coronary artery disease, and was termed percutaneous transluminal coronary angioplasty (PTCA) [16, 17]. Future advancements of this very procedure set out to revolutionize the treatment of patients with AMI. However, it was soon acknowledged that this novel PTCA procedure had one major limitation—acute coronary occlusion after angioplasty due to recoil or coronary dissection—which often led to severe complications including myocardial infarction or even death [18]. This limitation was recognized early on, and urged researchers to design a device that would have scaffolding effects and could thus prevent mechanical occlusion after balloon dilatation [19]. Accordingly, in 1986, the first generation of bare metal coronary stents—developed by Julio Palmaz and Richard Schatz—was launched as a solution for PTCA-related vessel complications. While the use of these coronary stents successfully reduced the rate of recoil and dissection-related coronary occlusions, it introduced a new risk of acute vessel occlusion, this time caused by thrombosis in the coronary stent [16, 20, 21].

During early animal experiments with coronary stents, Palmaz and Schultz observed that animals pretreated with aspirin, dipyridamole and dextran had a lower tendency for coronary clot formation [22]. On account of this, the first patients undergoing coronary stenting were treated unselectively with a broad regimen of antithrombotic medication, including both aspirin and dipyridamole, sulfinpyrazone (a cyclo-oxygenase-1 [COX-1] inhibitor), dextran and warfarin to prevent stent thrombosis. While this broad antithrombotic regimen was very successful in reducing ischemic complications (<1% subacute closure or myocardial infarction) at two weeks, the intensity of antithrombotic treatment needed to be deescalated due to significant increase in early bleeding complications (~10%) [23]. However, despite treatment with aspirin, dipyridamole and sulfinpyrazone on top of the existing anticoagulation regimen in patient undergoing percutaneous coronary intervention (PCI), stent thrombosis remained a feared complication (with an incidence up to 5%), driving the search for potent pharmacological options with an acceptable safety profile [19].

In subsequent years, ticlopidine, an adenosine diphosphate (ADP) receptor antagonist, was introduced. Combined with aspirin, ticlopidine significantly reduced the composite of cardiac death, myocardial infarction, coronary artery bypass grafting (CABG), or repeated angioplasty, as well as the rate of subacute stent thrombosis compared to anticoagulation therapy in patients undergoing PCI [24]. In the final years of the 20th century and the first decade of the 21st century, several other novel antiplatelet and anticoagulation agents—including glycoprotein IIb/IIIa receptor inhibitors (GPI), thrombin inhibitors and additional ADP receptor inhibitors, subsequently identified to selectively inhibit the ${\mathrm{P2Y}}_{12}$ receptor subtype—replaced ticlopidine. Each agent had its own unique pharmacokinetic and -dynamic profile as well as risk-benefit balance [25, 26, 27]. To date, a broad choice of antithrombotic treatment options exists to assist clinicians in reducing ischemic complications and improving outcomes in patients with AMI. Over the last decade, it has been increasingly recognized that bleeding complications in patients undergoing PCI for AMI are associated with increased risk of morbidity and mortality [28, 29]. On that account, the safety profile (i.e., bleeding risk) of existing antithrombotic agents has become crucial to the treatment of AMI.

## 3. The Requirement for Antithrombotic Therapy

The treatment objective of patients with STEMI is to attain early tissue reperfusion by recanalization of the infarct-related artery. Primary PCI has become the treatment of choice whenever it is readily available. Importantly, antithrombotic therapy—including antiplatelet and anticoagulation therapy—represents an essential component in the treatment of STEMI.

Periprocedural use of antithrombotic therapy is aimed at minimizing the risk of catheter-related complications including catheter thrombosis [30]. Additionally, it is increasingly recognized that antithrombotic therapy determines procedural success by establishing optimal coronary flow after stent implantation. However, to achieve successful epicardial recanalization, complete myocardial tissue reperfusion and an intact microvascular function of the myocardium is warranted. Indeed, approximately half of the STEMI patients develop downstream microvascular injury (shown by intramyocardial hemorrhage, microvascular obstruction and destruction documented on cardiac magnetic resonance imaging) despite successful primary PCI [31]. Moreover, approximately one third of STEMI patients develop major microvascular impairment defined as index of microcirculatory resistance >40 in the infarct-related perfusion territory after primary PCI [32]. Importantly, microvascular obstruction in STEMI patients after successful primary PCI has been significantly associated with a graded increase in mortality (per 1% absolute increase in microvascular obstruction; hazard ratio (HR) 1.14 [95% confidence interval (CI), 1.09–1.19], *p *< 0.0001) [33].

In addition to periprocedural safety and procedural success, the use of antithrombotic agents might extend an added protective effect when administered at an early (pre-hospital) time point. Early onset of antithrombotic effect may attenuate and even reverse the process of thrombus formation in a coronary artery prior to mechanical revascularization [34, 35]. Moreover, it has been suggested that early effective antithrombotic therapy may contribute to myocardial protection by decreasing microvascular obstruction through reducing distal thrombotic embolization. It has also been hypothesized that early effective antithrombotic therapy might reduce ischemia-reperfusion injury after successful revascularization [36, 37, 38, 39, 40, 41].

**Fig. 2.**
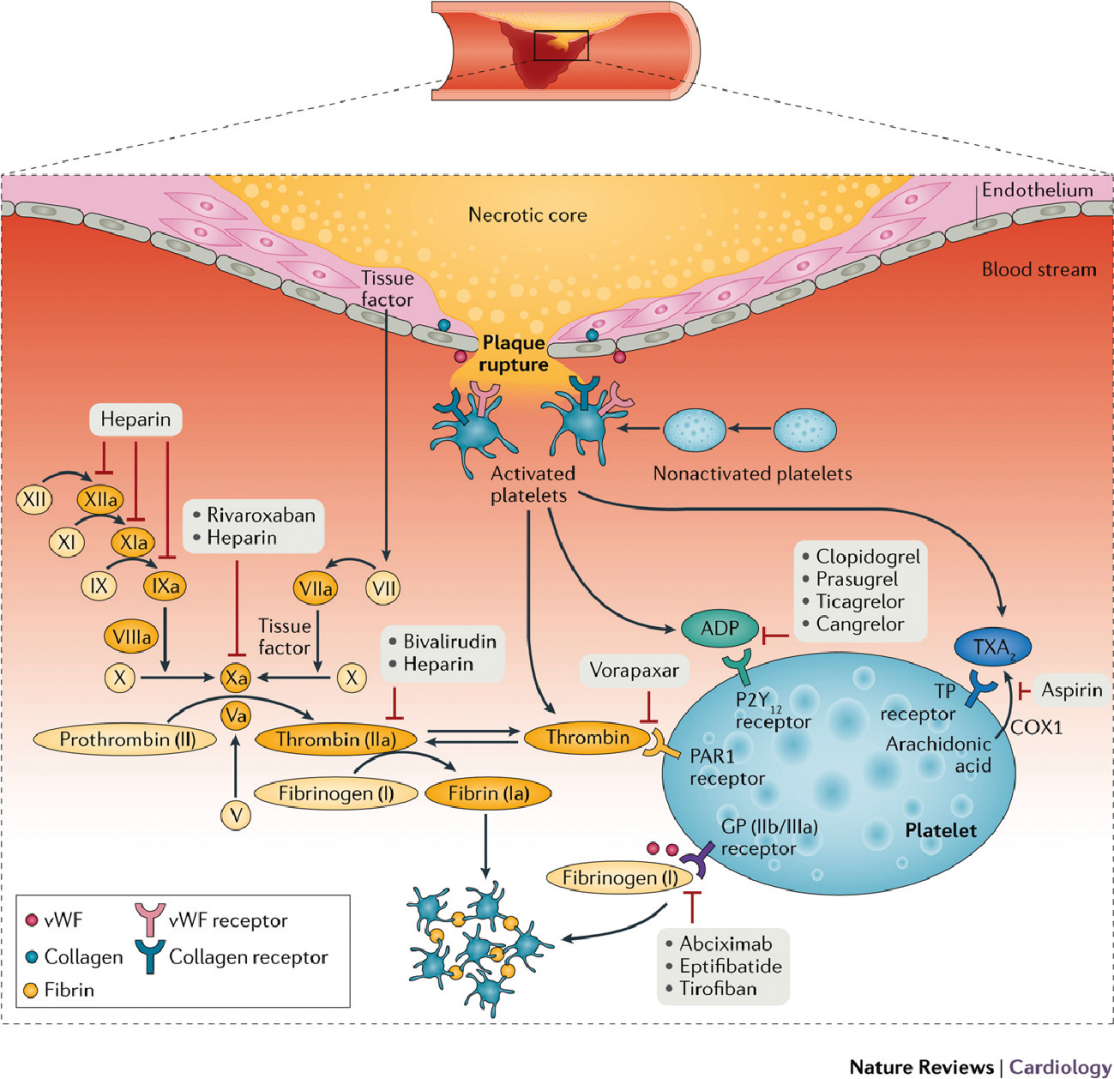
**Mechanism of thrombus formation during ST-segment elevation myocardial infarction, and targets of currently available antithrombotic agents**. After plaque rupture, adhesion of platelets to the subendothelium during the rolling phase is mediated by the interaction between the glycoprotein (GP) Ib/V/IX receptor complex located on the platelet surface and von Willebrand factor (vWF), and between collagen exposed at the site of vascular injury and platelet collagen receptors. Binding of collagen to these receptors triggers intracellular mechanisms that induce the release of activating factors, mainly thromboxane A2 (TXA2), ADP, and thrombin. These factors enhance the interactions among adherent platelets and promote further recruitment and activation of circulating platelets. Platelet activation by these mediators has as the final pathway the conversion of the platelet GP IIb/IIIa receptor, the main receptor mediating platelet aggregation, into its active form. Activated GP IIb/IIIa receptors bind to fibrinogen and vWF, leading to platelet aggregation and thrombus formation mediated by platelet–platelet interactions. Vascular injury also exposes subendothelial tissue factor, which forms a complex with factor VIIa and sets off a chain of events that culminates in formation of the prothrombinase complex. Prothrombin is converted to thrombin, which subsequently converts fibrinogen to fibrin, generating a fibrin-rich clot, and further activates platelets by binding to protease-activated receptors (PAR1) on the platelet membrane. However, only a modest amount of thrombin is produced as a result of the coagulation cascade, and the surface of activated platelets is the main source of circulating thrombin. Antiplatelet and anticoagulant agents work by inhibiting key receptors and factors involved in this cascade of events. COX, cyclooxygenase; TP, thromboxane prostanoid. *Reproduced from Franchi, F. et al. (2017) with permission from the authors (Dominick J. Angiolillo)* [42].

To date, several antithrombotic treatment options exist, targeting mediators and receptors involved with thrombosis (Fig. 2, Ref. [42]). The following section provides details on antiplatelet agents, both intravenous and oral, as well as intravenous anticoagulation agents used in the early management of patients presenting with STEMI.

## 4. Antiplatelet Therapy

The current recommended antiplatelet treatment for patients with STEMI who are planned to undergo primary PCI involves the combination use of the COX-1 inhibitor aspirin and an ADP receptor antagonist targeting the ${\mathrm{P2Y}}_{12}$ receptor (ticagrelor, prasugrel, clopidogrel or cangrelor), also referred to as dual antiplatelet therapy (DAPT) [7, 43, 44]. The first generation thienopyridine ticlopidine is no longer being used for the treatment of STEMI, due to rare but severe side effects such as neutropenia and is therefore beyond the scope of this review [45]. The intravenous antiplatelet glycoprotein IIb/IIIa inhibitors (GPI) abciximab, eptifibatide, and tirofiban are described later in a separate section.

### 4.1 Aspirin

Acetylsalicylic acid (aspirin) has been available since 1899 and was commonly used as a “pain killer” and anti-inflammatory drug. Its precise mechanism of action remained unclear until the beginning of the 1970s, when it was found to irreversibly inhibit the COX-1 enzyme, which is involved in the conversion of arachidonic acid into eicosanoids such as prostaglandins and thromboxane (${\mathrm{TXA}}_{2}$) [46]. The decreased synthesis of prostaglandins and ${\mathrm{TXA}}_{2}$ in platelets probably accounts for much of aspirin’s antithrombotic effect [47, 48, 49]. Orally administered aspirin (300–500 mg) is quickly absorbed and is responsible for >95% of the ${\mathrm{TXA}}_{2}$ synthesis, resulting in complete arachidonic acid-induced platelet aggregation inhibition within 20 minutes [50, 51, 52]. Intravenous administration provides fast and near complete inhibition of thromboxane synthesis within five minutes [51].

While aspirin has been available since 1899, it was not until the 1970s—supported by new insights into the pathophysiology of thrombosis, that the hypothesis was formed that aspirin might protect patients against thrombotic events [53]. One of the first landmark trials demonstrated the efficacy of aspirin in patients with confirmed arterial disease including coronary artery disease and a high risk of thrombotic events. Patients receiving long-term aspirin therapy showed a striking reduction of approximately 25% in their yearly risk of serious adverse vascular events [54, 55]. Published in 1988, the ISIS-2 was the first randomized clinical trial to demonstrate the efficacy of aspirin in reducing vascular mortality in AMI. The use of aspirin let to a highly significant 20% reduction in 5-week vascular mortality, which was similar to the 23% reduction facilitated by streptokinase treatment. When streptokinase and aspirin were combined, five-week vascular mortality was reduced by 40% [56]. Moreover, treatment with aspirin in this trial was not associated with a significant increase in intracerebral bleeding or transfusion-required bleeding.

Currently, both the American College of Cardiology Foundation (ACCF)/American Heart Association (AHA) and the European Society of Cardiology (ESC) guidelines recommend treatment with aspirin before primary PCI for patients with suspected STEMI, with latter recommending aspirin administration as early as possible [7, 43]. Aspirin loading dose can be given as an oral 150–300 mg dose of non-enteric-coated aspirin, or as a 250–500 mg intravenous dose.

### 4.2 Clopidogrel

Clopidogrel is a second-generation thienopyridine which succeeded the first-generation thienopyridine ticlopidine and was approved by the Food and Drug Administration (FDA) in 1997. It selectively and irreversibly inhibits the platelet ADP receptor, thereby preventing platelet activation and aggregation [57, 58]. The oral prodrug clopidogrel is metabolized by the liver into an active metabolite that binds and blocks the ${\mathrm{P2Y}}_{12}$ receptor [59, 60]. However, a large proportion (~85%) of the absorbed prodrug is promptly inactivated and excreted prior to hepatic metabolism. In healthy individuals, a single loading dose of 400 mg induces a significant inhibition of ADP-induced platelet aggregation of approximately 40% (compared to baseline platelet inhibition) within two hours of oral administration [61].

The randomized CURE trial (2001) was the first to review the efficacy of clopidogrel 300 mg loading dose on top of an aspirin 325 mg loading dose in patients presenting with non-ST-segment elevation acute coronary syndrome (NST-ACS) [25]. Clopidogrel led to a significant reduction in cardiovascular death, nonfatal myocardial infarction, or stroke at one year (9.3% versus 11.4%, relative risk [RR] 0.80 [95% confidence interval [CI], 0.72–0.90], *p *< 0.01). However, this benefit in ischemic outcomes came at the cost of an increase in TIMI major bleeding in the clopidogrel group (3.7% versus 2.7%, RR 1.38 [95% CI, 1.13–1.67], *p *< 0.01). The first PCI trial to show a beneficial effect of clopidogrel-pretreatment was the CREDO trial who randomized patients undergoing elective PCI to receive 300 mg clopidogrel loading dose or placebo 3 to 24 hours before PCI. Both groups received immediately after the PCI procedure 75 mg/d of clopidogrel and 325 mg/d of aspirin through day 28. A significant reduction in the combined endpoint death/MI/urgent TVR was seen in patients pretreated for at least 6 hours before PCI (5.8% pretreatment versus 9.4%, *p* = 0.05) but not in patients pretreated less than 6 hours before PCI [62]. Major bleeding did not differ between the pretreatment and no pretreatment arms regardless of the use of adjunct GPI.

Four years later, the efficacy of clopidgrel was reviewed in STEMI patients for the first time [63, 64]. The CLARITY trial examined clopidogrel (300 mg loading dose followed by a once-daily 75 mg maintenance dose for max. eight days) compared with a placebo in 3.491 STEMI patients treated with fibrinolytic therapy and who were planned to undergo angiography within 2–8 days [64]. Clopidogrel was associated with a 6.7% absolute reduction of the composite endpoint TIMI 0–1 flow in the infarct-related artery on angiography, death or myocardial infarction before angiography (15.0% versus 21.7%, *p *< 0.01). TIMI major and intracranial bleeding rates at 30 days were similar between groups. Respectively, the COMMIT trial examined the efficacy of clopidogrel (75 mg) versus a placebo in addition to aspirin (162 mg) for four weeks in over 45,000 patients with AMI who were managed conservatively [63]. They reported a significant reduction of the composite endpoint of death, reinfarction, or stroke in patients treated with clopidogrel (9.2% versus 10.1%, *p* = 0.002), again without an excess in fatal, transfusion-requiring, or cerebral bleeding. Interestingly, both the CLARITY and COMMIT trial found similar rates of protocol-defined major bleeding between groups, while the CURE trial reported higher bleeding rates for patients treated with clopidogrel. One possible explanation might be patient characteristics and duration of DAPT since the CURE trial included older patients and longer DAPT treatment duration (median of 9 months) compared to the COMMIT and CLARITY trials.

Despite the decrease in major adverse events facilitated by clopidogrel, stent thrombosis still occurred in approximately 2% of the treated STEMI patients, with detrimental effects on survival [65]. Importantly, pharmacodynamic and genetic studies have shown several drawbacks associated with the use of clopidogrel. In particular, there is broad inter-individual variability in response to clopidogrel which has shown to affect clinical outcomes, most importantly patients with impaired response are at increased risk of thrombotic complications [66, 67]. An overview of the epidemiology, diagnosis and clinical implication of clopidogrel resistance goes beyond the scope of this article but has been reviewed elsewhere [67, 68]. Moreover, slow gastro-intestinal absorption of clopidogrel, provoked by selective shunting of blood towards vital organs due to adrenergic activation, resulted in a delayed onset of antiplatelet effect [66, 69, 70, 71]. As a result, the onset of antiplatelet effect from clopidogrel is attenuated and therefore frequently insufficient at the time of primary PCI [66, 72]. To bridge these limitations, high loading doses of clopidogrel (e.g., 600 mg) have been tested and shown to enhance platelet inhibition compared to a 300 mg loading dose of clopidogrel [73]. However, despite the use of this regimen, platelet inhibition remains inadequate in many patients prompting the development of new ${\mathrm{P2Y}}_{12}$ inhibitors with a faster onset and more potent and predicable action.

To date, ${\mathrm{P2Y}}_{12}$ inhibitor treatment represents the mainstay of antithrombotic treatment and is recommended for the treatment of STEMI, before (or latest at the time of) PCI as a Class I indication [7, 43]. However, due to its pharmacodynamic limitations, clopidogrel use is reserved for patients in whom the use of more potent ${\mathrm{P2Y}}_{12}$ inhibitors (i.e., ticagrelor and prasugrel) is contraindicated, when these agents are not available, and is generally preferred in patients with an indication for chronic oral anticoagulation therapy. In these cases, a clopidogrel loading dose of 600 mg is recommended in patients with STEMI undergoing primary PCI. Of note, whether clopidogrel resistance affects outcomes in patients with an indication for oral anticoagulation receiving dual or triple antithrombotic therapy remains unknown.

### 4.3 Prasugrel

Prasugrel is a third generation thienopyridine, that was introduced in 2009. The pharmacology of prasugrel largely resembles that of clopidogrel. Like clopidogrel, prasugrel is orally administered as a prodrug, which is metabolized by the liver into an active metabolite that blocks the ${\mathrm{P2Y}}_{12}$ receptor. However, due to its more favorable pharmacokinetic profile, prasugrel is rapidly metabolized, resulting in prompt and potent platelet inhibition, which is more predictable and consistent than clopidogrel [74, 75, 76]. In healthy individuals and patients with stable coronary artery disease, a prasugrel loading dose of 60 mg achieved significantly greater platelet inhibition at one hour compared with 600 mg of clopidogrel (30% versus <5%, *p *< 0.0001) [77].

The efficacy of prasugrel compared to clopidogrel was assessed in the landmark TRITON-TIMI 38 trial [26]. Over 13,000 ACS patients (approximately 10,000 moderate-to-high risk unstable angina or NSTE-ACS patients, and 3500 STEMI patients) were allocated to receive either prasugrel (60 mg) or clopidogrel (300 mg) between randomization and one hour after PCI. Prasugrel compared with clopidogrel led to a significant reduction of the composite endpoint cardiovascular death, nonfatal myocardial infarction and nonfatal stroke at one year (9.9% versus 12.1%, hazard ratio [HR] 0.81 [95% CI, 0.73–0.90], *p *< 0.01). Moreover, prasugrel was associated with a significant reduction in stent thrombosis at 15 months (1.1% versus 2.4%, HR 0.48 [95% CI, 0.36–0.64], *p *< 0.001). However, a significant increase in TIMI major bleeding, including a significant increase in life-threatening bleedings, was observed in the prasugrel group (1.4% versus 0.9%, HR 1.52 [95% CI, 1.08–2.13], *p* = 0.01). This effect was mainly confined to elderly patients (>75 years), patients with previous cerebrovascular events (CVA) and patients with low body weight (<60 kg). The TRITON-TIMI STEMI sub analysis demonstrated an additional reduction in myocardial re-infarction (4.9% versus 7.0%, HR 0.70 [95% CI, 0.53–0.92], *p* = 0.01) and cardiovascular death (1.4% versus 2.4%, HR 0.61 [95% CI, 0.37–1.00], *p* = 0.04) at 30 days for patients treated with prasugrel [78]. In line with the main trial results, STEMI patients treated with prasugrel more frequently exhibited CABG-related TIMI major bleeding. However, non-CABG related TIMI major bleeding rates were similar between groups.

The superiority of prasugrel over clopidogrel was confirmed in a meta-analysis including 12 RCT’s and containing 14,701 STEMI patients undergoing primary PCI that were treated with prasugrel or clopidogrel [79]. Prasugrel therapy was associated with a relative risk reduction of RR 0.56 (95% CI, 0.43–0.73; *p *< 0.001) compared to clopidogrel in mortality, and a RR 0.71 (95% CI, 0.57–0.89; *p* = 0.002) reduction in MACE (NB: definitions used for MACE were sometimes trial-dependent), without a significant increase in TIMI major bleeding (RR 1.54 [95% CI, 0.64–3.71], *p* = 0.34).

Inherent to the oral administration route, prasugrel exhibits the same drawback as clopidogrel: a slow onset of action due to delayed gastro-intestinal absorption [80, 81, 82]. To overcome this limitation, alteration in time point of administration and modification of the tablets have been tested. Crushing oral tablets of prasugrel before administration was tested in pharmacokinetic and -dynamic studies in STEMI patients undergoing primary PCI. Crushed tablets compared to integral tablets led to an approximate 20% reduction in high platelet reactivity (defined as ${\mathrm{P2Y}}_{12}$ reactivity unit (PRU) ≥208) 30–45 minutes after administration [83, 84]. However, when investigated in a clinical trial, pre-hospital administration of crushed prasugrel tablets in STEMI patients failed to improve early myocardial reperfusion or clinical outcomes [85, 86].

Interestingly, prasugrel was never tested in patients treated with fibrinolysis in contrast to ticagrelor [87].

Ultimately, prasugrel is one of the most potent oral ${\mathrm{P2Y}}_{12}$ inhibitors currently available with results suggesting better outcomes compared with clopidogrel and is therefore recommended with a class I indication for patients presenting with STEMI, administered before or latest at time of primary PCI [7, 43]. Notably, prasugrel use is contraindicated in patients with a history of CVA, and its use is generally not recommended in elderly patients (>75 years) or patients with lower body weight (<60 kg), unless a maintenance dose-reduction to 5 mg is being made.

### 4.4 Ticagrelor

Two years after prasugrel received FDA approval, the cyclopentyltriazolopyrimidine ticagrelor, a novel ADP analogue, was approved. Ticagrelor directly but reversibly inhibits the ${\mathrm{P2Y}}_{12}$ receptor. In contrast to clopidogrel and prasugrel, ticagrelor is not a prodrug and does not require metabolic activation, although 30% of the effects of the drug derive from an hepatic-derived metabolite. Moreover, ticagrelor and its metabolite both actively inhibit ADP mediated platelet activation of the ${\mathrm{P2Y}}_{12}$ pathway by reversibly binding to a distinct site of the receptor [88, 89]. Consequently, the antiplatelet effect of ticagrelor is faster, more potent, and more predictable than the antiplatelet effect constituted by clopidogrel. In stable patients, near-complete platelet inhibition effect is achieved two hours after administration [88, 90, 91, 92, 93]. In patients presenting with ACS, ticagrelor provides stronger platelet inhibition one hour post-PCI compared with clopidogrel [94, 95]. Ticagrelor additionally provides a non‑${\mathrm{P2Y}}_{12}$-mediated increase in adenosine plasma levels due to inhibition of cellular uptake [96]. It is not clear how this contributes to the observed general benefits of the drug but has been associated with the side-effect of dyspnea in up to one out of five patients [27, 97].

The efficacy and safety of ticagrelor compared with clopidogrel was established in the PLATO trial [27]. Over 18,000 ACS patients were randomized to receive either ticagrelor (180 mg loading dose followed by a maintenance dose of 90 mg twice daily) or clopidogrel (300–600 mg loading dose followed by a maintenance dose of 75 mg) in addition to aspirin. Ticagrelor significantly reduced the primary composite endpoint of vascular death, myocardial infarction, or stroke at one year (9.8% versus 11.7%, HR 0.84 [95% CI, 0.77–0.92], *p *< 0.01). Moreover, all-cause death and definite or probable stent thrombosis rates were significantly reduced in the ticagrelor group. Nonetheless, ticagrelor treatment was associated with a significant increase in non-CABG related TIMI major bleeding (4.5% versus 3.8%, HR 1.19 [95% CI, 1.02–1.38], *p* = 0.03), including more fatal intracranial bleeds. However, the overall major bleeding rates were similar between groups (11.6% versus 11.2%, HR 1.04 [95% CI, 0.95–1.13], *p* = 0.43). Remarkably, in the STEMI subgroup of the PLATO trial (n = 7544), ticagrelor treatment did not result in a significant reduction of the composite endpoint of myocardial infarction, stroke, or cardiovascular death (10.8% versus 9.4%, HR 0.87 [95% CI, 0.75–1.01], *p* = 0.07) [95]. However, patients treated with ticagrelor did have significantly lower rates of myocardial re-infarction, stent thrombosis and total mortality, without a significant increase in PLATO or TIMI-classified major bleeding rates.

These results on the efficacy of ticagrelor were supported by a meta-analysis including STEMI patients undergoing primary PCI that were treated with ticagrelor (n = 4031), or clopidogrel (n = 9234) [79]. Ticagrelor treatment compared with clopidogrel treatment significantly reduced the risk of MACE, mortality, and stent thrombosis, respectively (OR 0.49 [95% CI, 0.27–0.89], *p* = 0.02; OR 0.80 [95% CI, 0.66–0.98], *p* = 0.03, and OR 0.62 [95% CI, 0.43–0.89], *p* = 0.01).

Like prasugrel, administration of crushed tablets of ticagrelor in STEMI patients facilitates faster gastro-intestinal absorption, and results in higher levels of platelet inhibition at the time of primary PCI (98,99). However, despite this improvement in pharmacodynamic profile, high platelet reactivity during the first hour of administration is seen in 35–77% of patients [98, 99]. The potential of early pre-hospital administration of oral ${\mathrm{P2Y}}_{12}$ inhibitors to overcome the high platelet reactivity observed during primary PCI was investigated in the ATLANTIC trial [100]. STEMI patients were randomized to receive a loading dose of ticagrelor either at first medical contact in the ambulance, or after arrival at the hospital but before undergoing primary PCI. Pre-hospital ticagrelor administration failed to show improvement in markers of early myocardial reperfusion, although a trend was seen for improvement in complete ST-segment resolution in patients who had not received morphine. Moreover, there was an indication that pre-hospital administration of ticagrelor might reduce post-procedural stent thrombosis rates since all stent thrombosis events occurred within the first 24 hours in the in-hospital group and the difference remained significant in favor of pre-hospital ticagrelor administration until 30 days. Of note, the median time difference between the two groups consisted of 30 minutes, a time-window in which a possible clinical benefit of pretreatment—in view of the pharmacokinetics of ticagrelor—might not have had the chance to present itself.

Moreover, ticagrelor was tested in a phase 2 study with lytics and did not significantly reduce the incidence of cardiovascular events after fibrinolytic therapy when compared with clopidogrel and thus has not been approved for this indication [87].

Prasugrel and ticagrelor are the most potent oral ${\mathrm{P2Y}}_{12}$ inhibitors currently available and have received a Class I recommendation in the treatment of patients with STEMI [7, 43]. Despite differences in the metabolism of the two agents, the pharmacodynamic profiles appear to be comparable, including the early response after loading dose administration [101, 102]. While compelling evidence has demonstrated the superiority of prasugrel and ticagrelor over clopidogrel in the treatment of STEMI patients, there is less evidence demonstrating the superiority of prasugrel or ticagrelor over the other.

The randomized PRAGUE-18 trial assessed the efficacy of prasugrel versus ticagrelor in STEMI patients, but was prematurely terminated due to futility, with only 45% inclusions of the estimated sample size [97]. The analysis of the available data showed similar incidences of the composite of death, reinfarction, urgent target vessel revascularization, stroke or serious bleeding requiring transfusion at seven days for both the ticagrelor and prasugrel treated patients (4.0% versus 4.1%, odds ratio [OR] 0.98 [95% CI, 0.55–1.73], *p* = 0.94). Furthermore, TIMI major and Bleeding Academic Research Consortium (BARC) type ≥3 rates at 30 days were similar between groups in this study.

The randomized ISAR-REACT 5 trial investigated ticagrelor compared with prasugrel in over 4000 patients with ACS [103]. The occurrence of the composite endpoint of death, myocardial infarction and stroke at one year was significantly lower in the prasugrel treated group compared with ticagrelor at one year (9.3% versus 6.9%, HR 1.36 [95% CI, 1.09–1.70], *p *< 0.01). This effect was primarily driven by a reduction in nonfatal myocardial infarction, although there was an additional numerically lower all-cause mortality. Importantly, major bleeding (BARC type ≥3) rates were similar between groups (5.4% versus 4.5%, HR 1.12 [95% CI, 0.83–1.51], *p* = 0.46). The subgroup analysis from ISAR-REACT 5 in STEMI patients (n = 1653), showed a numerical, yet not significant, difference in the occurrence of the primary endpoint for patients treated with prasugrel (10.1% versus 7.9%, HR 1.31 [95% CI, 0.95–1.82], *p* = 0.10) [104]. The STEMI guidelines have not been updated since ISAR-REACT 5 and do not recommend a preference regarding the use of ticagrelor or prasugrel for the treatment of STEMI, as opposed to the NSTE-ACS guideline which favors prasugrel based on the results of the ISAR-REACT trial [7, 43, 105].

### 4.5 Cangrelor

The first and currently only available intravenous ${\mathrm{P2Y}}_{12}$ inhibitor is cangrelor, which became available in 2013. Like the oral ${\mathrm{P2Y}}_{12}$ inhibitor ticagrelor, cangrelor reversibly inhibits the ${\mathrm{P2Y}}_{12}$ receptor without prior hepatic metabolism. Cangrelor has a predictable, linear pharmacokinetic profile, with a $T_{\text{max}}$ of two minutes for the standardized dose of 30 μg/kg bolus followed by a 4 μg/kg/min infusion [106]. The onset of platelet inhibition effect follows almost immediately after bolus administration [107, 108]. The rapid inactivation of circulating cangrelor (3–6 minutes) causes a swift offset of antiplatelet effect returning to baseline levels within 30–60 minutes after drug discontinuation, and therefore requires timely co-administration with an oral ${\mathrm{P2Y}}_{12}$ inhibitor [106, 109, 110]. Since cangrelor directly inhibits the active metabolite of thienopyridines from binding to the ${\mathrm{P2Y}}_{12}$ receptor, timely co-administration of ticagrelor is recommendable over clopidogrel or prasugrel [107, 111, 112, 113]. Due to its faster onset of antiplatelet effect compared with oral ${\mathrm{P2Y}}_{12}$ inhibitors, cangrelor represents an alternative to oral agents for the acute phase of STEMI [111, 114].

In a series of three large landmark trials, the CHAMPION researchers investigated the use of cangrelor in different clinical settings. The first CHAMPION trial (CHAMPION PCI) assessed the efficacy of cangrelor compared with a 600 mg clopidogrel loading dose 30 minutes before the start of PCI in patients with ACS [115]. No significant difference was found in the occurrence of the composite of all-cause mortality, myocardial infarction, or ischemia driven revascularization at 48 hours between groups (7.5% versus 7.1%, OR 1.05 [95% CI, 0.88–1.24], *p* = 0.59). TIMI major bleeding was borderline increased in the cangrelor group (3.6% versus 2.9%, OR 1.26 [95% CI, 0.99–1.60], *p* = 0.06).

The second CHAMPION trial (CHAMPION PLATFORM) investigated the efficacy of cangrelor versus a placebo as an adjunctive treatment at the time of PCI in ACS patients excluding patients with STEMI [116]. This trial was terminated prematurely because the likelihood of treatment benefit for the primary composite endpoint of death, myocardial infarction or ischemia-driven revascularization within 48 hours was considered low. Although the primary endpoint was similar between groups (7.0% versus 8.0%, OR 0.87 [95% CI, 0.71–1.07], *p* = 0.17), subsequent analyses indicated that stent thrombosis and all-cause mortality were significantly less frequent in the cangrelor group compared with a placebo (stent thrombosis; 0.2% versus 0.6%, OR 0.31 [95% CI 0.13–0.83], *p* = 0.02, and mortality; 0.2% versus 0.7%, OR 0.33 [95% CI, 0.11–0.85], *p* = 0.02). However, this effect was hampered by a significant increase in bleeding events according to the Acute Catheterization and Urgent Intervention Triage strategy (ACUITY) bleeding classification driven by increase in access site hematomas in the cangrelor group. However, no increase in GUSTO (according to the Global Use of Streptokinase and t-PA for Occluded Coronary Artery) and TIMI (Thrombolysis in Myocardial Infarction) major bleedings were seen.

In the third and final CHAMPION trial (CHAMPION PHOENIX), the efficacy of cangrelor was assessed compared with clopidogrel (300–600 mg) in patients undergoing either urgent or elective PCI [117]. This study found a significant reduction in the occurrence of death, myocardial infarction, ischemia-driven revascularization, or stent thrombosis at 48 hours for patients treated with cangrelor (4.7% versus 5.9%, OR 0.78 [95% CI, 0.66–0.93], *p *< 0.005). This effect was mainly driven by the reduction in myocardial infarction. Importantly, the occurrence of perprocedural stent thrombosis was significantly reduced in the cangrelor group (0.6% versus 1.0%, OR 0.65 [95% CI, 0.42–0.99], *p* = 0.04). The ischemic benefit of cangrelor was independent of clinical presentation, and seen in elective PCI, ACS and STEMI patients. There was no increase in severe or life-threatening GUSTO bleeding within the first 48 hours (0.2% versus 0.1%, OR 1.50 [95% CI, 0.53–4.22], *p* = 0.44). However, ACUITY bleeding rates were significantly higher for patients treated with cangrelor. This was primarily caused by more access site related hematomas (12.0% versus 9.3%, OR 1.34 [95% CI, 1,12–1.59], *p* = 0.001).

At the time of the CHAMPION trials, cangrelor showed the potential to reduce ischemic complications in STEMI patients with however an increase in ACUITY defined major bleedings driven by access site hematomas but without increase of TIMI or GUSTO major bleedings. A pooled analysis of the 3 CHAMPION trials demonstrated cangrelor’s efficacy in reducing ischemic adverse events compared to clopidogrel in patients undergoing PCI irrespective of GPI administration [118]. However, GPI use was associated with substantially higher bleeding rates regardless to cangrelor or clopidogrel concomitant treatment. To date, there has been no direct comparison of cangrelor and the more potent ${\mathrm{P2Y}}_{12}$ inhibitors ticagrelor and prasugrel, combined with modernized interventional techniques.

The routine use of cangrelor is therefore not recommended in the current guidelines. However, cangrelor may be considered in ${\mathrm{P2Y}}_{12}$ inhibitors naïve patient at the time of PCI or who are unable to absorb oral agents (i.e., patients presenting with cardiogenic shock or circulatory arrest) and is endorsed by the current guidelines with a Class IIb recommendation [7].

## 5. Anticoagulation Therapy

Anticoagulant therapy is an essential prerequisite for primary PCI to avoid thrombotic complications. Currently available agents include unfractionated heparin (UFH), the low molecular weight heparin enoxaparin and the direct thrombin inhibitor bivalirudin. As the use of the direct factor Xa inhibitor fondaparinux has been associated with significant rates of catheter thrombosis, it is no longer recommended in the treatment during primary PCI, and it is therefore not separately discussed in this review.

### 5.1 Unfractionated Heparin

UFH has been used as an anticoagulation agent since the beginning of the 20th century. Intravenously administered UFH binds to circulating antithrombin, which is an important endogenous inhibitor of the coagulation cascade [119]. By binding to antithrombin, UFH inactivates the formation of thrombin and several coagulation factors, including Xa, IXa, Xia and XIIa. Moreover, UFH enhances endogenous fibrinolysis [120]. A single bolus of UFH results in an almost immediate anticoagulant effect. However, UFH use has several drawbacks: Aside from antithrombin, UFH additionally binds to circulating plasma proteins, which leads to significant variability in inter-individual response bioavailability and requires pharmacodynamic monitoring via activated clotting time or activated partial thromboplastin time to guide UFH dosage during PCI [121, 122]. Moreover, UFH can prevent the formation of new thrombin, but is unable to interact with thrombin that is already cloth-bound [123]. Lastly, in rare cases UFH can sometimes trigger a severe and potentially lethal condition called heparin-induced thrombocytopenia [124].

UFH has been an essential part of PCI for several decades. The efficacy of UFH has been established in comparison to other antithrombotic therapies, including bivalirudin, fondaparinux and low molecular weight heparin (LMWH) enoxaparin. The randomized OASIS-6 trial investigated the early use of UFH compared with fondaparinux as an adjunctive to PCI in 12,000 patients with ACS [125]. While fondaparinux therapy was associated with a reduction of death or reinfarction at 30 days in the overall cohort, there was a trend towards increased risk of death or reinfarction for patients undergoing primary PCI (PCI group: UFH 5.1% versus 6.1%, OR 1.20 [95% CI, 0.91–1.57], no-PCI group: UFH 13.8% versus 11.5%, OR 0.82 [95% CI, 0.66–1.02], *p*-interaction = 0.03). Moreover, in STEMI patients undergoing primary PCI, UFH therapy was associated with significantly less pharmacological bailout therapy with intravenous glycoprotein IIb/IIIa inhibitors, less guiding catheter thrombosis and less coronary complications compared with fondaparinux therapy.

To date, UFH remains an important component for the pharmacological treatment of STEMI in patients undergoing primary PCI. The dosing of UFH has undergone significant adjustment throughout the history of PCI. Currently, routine use of UFH during primary PCI (70–100 U/kg bolus, and 50–70 U/kg when GPI use is intended) holds a Class I recommendation [7]. Patients who received an initial UFH bolus prior to primary PCI are recommended to receive an additional dose of 2000–5000 U during PCI, to achieve an ACT of 250–300 seconds (HemoTec) or 300–350 seconds (Hemochron) [126]. There is convincing evidence that early UFH administration in a pre-hospital setting improves outcomes and this strategy has been adapted in several local STEMI protocols [127, 128].

### 5.2 Low-Molecular-Weight Heparin

The limitations related to UFH, including the unpredictability of its effect and the risk of heparin induced thrombocytopenia, have prompted the search for other anticoagulation agents, including low molecular weight heparins (LMWH) [129]. While several LMWH agents have been developed, enoxaparin is the most extensively tested once in the setting of PCI. Enoxaparin is a subcutaneously administered LMWH that specifically targets coagulation factors Xa and IIa [119]. The low molecular weight of enoxaparin translates into a reduced binding capacity of heparin to circulating proteins, resulting in a more predictable, dose-dependent profile without the need for pharmacodynamic monitoring. Moreover, enoxaparin is able to inactivate surface bound coagulation factors, increasing its efficacy, making enoxaparin a promising alternative for UFH [123].

The randomized ATOLL trial reviewed the efficacy of enoxaparin (administered as a 0.5 mg/kg bolus) as a replacement of UFH in STEMI patients undergoing primary PCI [130]. The primary composite endpoint of death, complications of myocardial infarction, procedure failure or Safety and Efficacy of Enoxaparin in Percutaneous Coronary Intervention Patients, an International Randomized Evaluation (STEEPLE) classified major bleeding at 30 days was numerically, but not significantly, lower in the enoxaparin group (28% versus 34%, RR 0.83 [95% CI, 0.68–1.01], *p* = 0.06). However, enoxaparin significantly reduced the main secondary endpoint which was a composite of death, recurrent ACS, or urgent revascularization (7% versus 11%, RR 0.59 [95% CI, 0.38–0.91], *p* = 0.015). Major bleeding rates at 30 days were similar between groups (5% versus 5%, RR 0.92 [95% CI, 0.51–1.66], *p* = 0.79).

Interestingly, a meta-analysis reviewing the ATOLL trial and three other RCT’s and including 5585 STEMI patients, reported a significant reduction in the rate of MI (OR 0.74 [95% CI, 0.60–0.90], *p *< 0.01) and death (OR 0.74 [95% CI, 0.56–0.97], *p* = 0.03) for patients who were treated with enoxaparin compared with UFH, without a significant increase in major bleeding (OR 0.81 [95% CI, 0.53–1.24], *p* = 0.33) [131].

To date, enoxaparin has a limited role in the treatment of STEMI. Based on the primary endpoint results from the ATOLL trial, the American guidelines refrain from any recommendation concerning the use enoxaparin [43]. In contrast, the European guidelines have based their recommendation on the secondary endpoint results from the same trial, and advise to consider enoxaparin in patients undergoing primary PCI (Class IIa recommendation) [7].

### 5.3 Bivalirudin

Several years after the development of enoxaparin, the synthetic polypeptide bivalirudin was introduced as a new potential substitute of UFH. Bivalirudin is a small polypeptide that directly but reversibly inhibits thrombin by binding to both its active site and its fibrinogen binding site [132, 133]. The pharmacokinetic profile of bivalirudin is more predictable than UFH, and therefore requires no pharmacodynamic monitoring during bivalirudin treatment.

The efficacy of bivalirudin has been extensively investigated in dedicated trials. Early STEMI trials reported a promising signal of decrease in ischemic complications and major bleeding in patients treated with bivalirudin compared with UFH [134, 135, 136]. However, more recently conducted trials provided other insights on the use of bivalirudin [137].

The HORIZONS-AMI trial (published in 2008) investigated the efficacy and safety of bivalirudin versus UFH plus routine GPI in 3602 STEMI patients undergoing primary PCI [135]. Bivalirudin treatment compared with UFH plus routine GPI was associated with a significant reduction of net adverse clinical events (NACE) at 30 days (9.2% versus 12.1%, RR 0.76 [95% CI, 0.63–0.92], *p* = 0.005), primarily attributable to a significant lower rate of major bleeding (4.9% versus 8.3%, RR 0.60 [95% CI, 0.46–0.77], *p *< 0.001). Surprisingly, bivalirudin treatment was also associated with an increase in acute stent thrombosis (1.3% versus 0.3%, *p *< 0.001). This effect was attenuated in patients who received a bolus of UFH before bivalirudin treatment. Five years later, the EUROMAX trial reported similar results to the HORIZONS-AMI trial [136]. However, the EUROMAX trial was conducted in a more contemporary setting than the HORIZONS-AMI trial, characterized by the upstream (pre-hospital) use of bivalirudin and no routine GPI administration. 2218 STEMI patients were randomized to bivalirudin or UFH/LMWH treatment prior to primary PCI. Patients treated with bivalirudin had a reduced risk of death or non-CABG related TIMI major bleeding compared with patients who received UFH/LMWH (5.1% versus 8.5%, RR 0.60 [95% CI, 0.54–0.96], *p* = 0.001), but again with an increased risk of acute stent thrombosis (1.1% versus 0.2%, RR 6.11 [95% CI, 1.37–27.24], *p* = 0.007).

Remarkably, the ischemic benefit of bivalirudin compared with UFH in 1812 patients undergoing primary PCI was completely absent in the HEAT-PPCI trial that was reported one year later [137]. The composite endpoint of all-cause mortality, CVA, reinfarction, or unplanned target lesion revascularization at 28 days was significantly more common in patients treated with bivalirudin, mainly driven by a higher rate of reinfarction in the bivalirudin group (bivalirudin 8.7% versus 5.7%, RR 1.52 [95% CI, 1.09–2.13], *p* = 0.01). Importantly, the risk of acute stent thrombosis was again significantly increased in patients that received bivalirudin.

In 2015, the MATRIX trial compared bivalirudin with or without post-PCI infusion to UFH in patients with ACS who were anticipated to undergo PCI [138]. Patients additionally received a ${\mathrm{P2Y}}_{12}$ inihibitor and UFH. Bivalirudin treatment was not associated with a significant reduction in MACE compared to UFH (10.4% versus 10.9%, RR 0.94 [95% CI, 0.81–1.09], *p* = 0.44). Moreover, a post-PCI infusion of bivalirudin did not significantly decrease the rate of urgent target-vessel revascularization, definite stent thrombosis, or net adverse clinical event (11.0% versus 11.9%, RR, 0.91 [95% CI 0.74–1.11], *p* = 0.34).

Overall, bivalirudin has been extensively reviewed in large-scale clinical trials conducted in different clinical settings over the years. A recently published meta-analysis comparing bivalirudin to UFH concluded that bivalirudin treatment significantly reduces major bleeding, but at cost of a significant increase in acute stent thrombosis [139]. These results have contributed to the continuing discussion about the value of bivalirudin in the setting of primary PCI. This ongoing debate is reflected in the differences between the European and American guidelines. These differences might also be contributable to the timing of guideline publications, as the ACCF/AHA guideline was published before the results from the EUROMAX and HEAT-PPCI trials were released. According to the ESC guideline, the use of bivalirudin is endorsed in patients with heparin-induced thrombocytopenia with a Class I recommendation, but receives only a Class IIa recommendation for its routine use [7]. In contrast, according to the ACC guidelines, bivalirudin is recommended with a Class I indication for periprocedural use with or without prior treatment with UFH. Moreover, bivalirudin is preferred over UFH in the ACC guidelines in patients with a high risk of bleeding (Class IIa) [43].

## 6. Glycoprotein IIb/IIIa Inhibitor Therapy

Currently available GPI’s include abciximab, tirofiban, and eptifibatide. The common target of all GPI’s is the final pathway of platelet aggregation. Early trials have demonstrated an important ischemic benefit of GPI therapy in ACS patients but with an additional unwanted significant increase in major bleeding. Interestingly, with the development of novel antithrombotic agents, more resent trials have shown less ischemic benefit with the routine use of GPI’s. Consequently, routine GPI use has fallen out of favor. GPI is currently reserved as a bailout strategy during no-reflow after PCI or when thrombotic complications occur [7, 126].

### 6.1 Abciximab

Abciximab was the first GPI that became available for clinical use in the 1990s. Abciximab is a human-murine chimeric antibody that irreversibly inhibits the binding capacity of the glycoprotein IIb/IIIa receptor to fibrinogen [140, 141]. Due to its high affinity for the glycoprotein IIb/IIIa receptor, a bolus injection of abciximab facilitates complete inhibition of all glycoprotein IIb/IIIa receptors within 30 minutes of administration [142]. The offset of antiplatelet effect however, may take several days to weeks [143].

The use of abciximab as an adjunctive to antithrombotic therapy in AMI patients has been extensively investigated over the years [144, 145, 146, 147, 148]. The first trials reported higher rates of early myocardial reperfusion and a significant reduction of ischemic complications in patients who were routinely treated with abciximab during PCI. However, this effect was accompanied by an increase in major (mostly arterial access site related) bleeding. Importantly, these trials were all conducted in a time when balloon angioplasty was the standard of care treatment for AMI, and DAPT with aspirin and clopidogrel had not yet been introduced.

In 2009, the randomized BRAVE-3 trial investigated the efficacy of abciximab after the introduction of clopidogrel for the treatment of STEMI [149]. Consecutive STEMI patients were randomized to receive abciximab (0.25 mg/kg bolus followed by an infusion of 0.125 μg/kg) or placebo pre-PCI in addition to clopidogrel and aspirin. Interestingly, in the contemporary setting of the BRAVE-3 trial, the significant benefit of abciximab in terms of death, recurrent myocardial infarction, stroke or urgent revascularization reported in earlier trials seemed no longer present (5.0% versus 3.8%, RR 1.30 [95% CI, 0.70–2.60], *p* = 0.40). However, no increase in TIMI major bleeding complications were reported either (1.8% versus 1.8%, *p* = 0.99).

The upstream use of abciximab in patients suspected of STEMI has been evaluated in several clinical trials with conflicting results [146, 150, 151, 152, 153]. In any case, a clear benefit of abciximab use in a contemporary setting has not been demonstrated.

### 6.2 Eptifibatide 

Eptifibatide is a small, cyclic hepapeptide, that was developed during the same period as tirofiban. Eptifibatide inhibits the GP IIb/IIIa receptor in a highly selective but reversible manner [154, 155]. After intravenous administration, a bolus of eptifibatide results in fast and effective platelet inhibition [156, 157]. The level of platelet inhibition is maintained over the course of a constant infusion of low dose eptifibatide. After discontinuation, the offset of antiplatelet effect occurs within four hours [156, 158, 159].

During the second half of the 1990s, two large landmark trials assessed the efficacy of eptifibatide in patients undergoing PCI [160, 161]. The IMPACT-II trial included over 4000 patients undergoing elective, urgent or emergency coronary intervention. Patients were allocated to a placebo treatment, or a 135 μg/kg bolus of eptifibatide followed by either a 0.5 μg/kg or a 0.75 μg/kg infusion [160]. Study medication was started 10–60 minutes before coronary procedure, and continued for 24 hours. Patients treated with eptifibatide 135/0.5 regimen had significantly lower rates of the composite of death, myocardial infarction, unplanned surgical or percutaneous coronary intervention at 30 days compared with the placebo group, without an increase in major bleeding.

The PERSUIT trial randomized approximately 11,000 NSTEMI patients to eptifibatide (followed by a high or low dose infusion) or placebo [161]. Notably, in this trial the eptifibatide infusion was continued up to 72 hours (or 96 hours if patients underwent PCI). Concomitant antithrombotic therapy included 325 mg of aspirin and a bolus injection of UFH followed by 1000 U/hour infusion. Eptifibatide was associated with a significant 1.5% reduction in death and non-fatal myocardial infarction at 30 days compared to placebo. However, TIMI major bleeds were significantly increased in the epitifibatide group (major: 10.6% versus 9.1, *p* = 0.02) as well as GUSTO moderate to severe bleeds (severe: 1.5% versus 0.9%, *p *< 0.001).

### 6.3 Tirofiban

Approximately one decade after the development of abciximab, a new GPI agent tirofiban was introduced for the treatment of STEMI. Tirofiban is a highly selective, non-peptide tyrosine derivative that was approved for clinical use in the year 2000. Similar to the working mechanism of abciximab, tirofiban competitively inhibits the glycoprotein IIb/IIIa receptor from binding to fibrinogen [141, 162]. Intravenous administration of tirofiban initiates a dose-dependent near complete antiplatelet inhibition effect within 15 minutes of administration [114, 163, 164]. The offset of antiplatelet effect takes approximately three hours following discontinuation.

The PRISM trial assessed the efficacy of a low-dose tirofiban (0.6 μg/kg bolus followed by 0.15 μg/kg infusion) compared with a standardized UFH regimen in over 3200 ACS patients [165]. All patients additionally received 300–325 mg of aspirin. Routine low-dose tirofiban treatment was associated with a significantly lower occurrence of the composite of death, reinfarction and refractory ischemia at 48 hours compared with UFH (3.8% versus 5.6%, RR 0.67 [95% CI, 0.48–0.92], *p* = 0.01). TIMI major bleeding rates were similar between groups (0.4% versus 0.4%).

A higher bolus dose of tirofiban (10 μg/kg) was investigated compared with a placebo in the RESTORE trial, which included over 2200 patients with ACS [166]. The trial’s primary endpoint was the composite of death from any cause, myocardial infarction, coronary bypass surgery due to angioplasty failure or recurrent ischemia, repeat target-vessel angioplasty for recurrent ischemia, and insertion of a stent due to actual or threatened abrupt closure of the dilated coronary artery at 30 days. There was no significant difference in the occurrence of the primary endpoint (10.3% versus 12.2%, *p* = 0.16) or in the occurrence of TIMI major bleeding (2.4% versus 2.1%, *p* = 0.66) between groups. However, the primary composite endpoint was significantly reduced in the tirofiban group at seven days (7.6% versus 10.4%, *p* = 0.02), indicating that tirofiban treatment might protect against early ischemic complications.

The STRATEGY trial was the first landmark trial that investigated the use of high bolus dose of tirofiban (25 μg/kg) combined with the use of a first generation sirolimus drug eluting stent (DES), compared to abciximab with a bare metal stent [167]. This trial demonstrated that the use of a high dose tirofiban as adjunctive to PCI with a first-generation DES resulted in a significant reduction of the occurrence of the composite of death, non-fatal myocardial infarction, stroke or binary restenosis at 8 months (19% versus 50%, HR 0.33 [95% CI, 0.18–0.60], *p *< 0.01), without any increase in TIMI major bleeding (1% versus 2%, *p* = 0.99).

The efficacy of upstream tirofiban use was reviewed in the ON-TIME 2 trial [34]. 984 patients suspected of STEMI were managed by the emergency medical service and received a high dose bolus of tirofiban or placebo, in addition to the standard treatment regimen (5000 IU of UFH, 500 mg i.v. aspirin and 600 mg clopidogrel). Patients that had received tirofiban showed a significant reduction in residual ST-segment deviation before PCI (10.9 ± 9.2 mm versus 12.1 ± 9.4 mm, *p* = 0.028), and an approximate 7% reduction in death, reinfarction, urgent target vessel revascularization or blinded bailout (26.0% versus 32.9%, *p* = 0.02). Importantly, this ischemic benefic came without a significant increase in TIMI major bleeding.

Unfortunately, clinical data on the use of upstream tirofiban in a more contemporary setting with improved interventional techniques and the potent ${\mathrm{P2Y}}_{12}$ inhibitors ticagrelor and prasugrel is currently limited. The pharmacodynamic FABOLUS FASTER trial might initiate a newfound interest in tirofiban as a treatment option for STEMI patients [114]. In this trial, patients were randomized to receive either high dose tirofiban (infusion for two hours), cangrelor (infusion for two hours), ticagrelor (180 mg, chewed) or ticagrelor (180 mg, integral) directly before primary PCI. A bolus of tirofiban resulted in a significant near-complete reduction of high platelet reactivity (defined as platelet aggregation >59% at LTA after ADP 20 μmol/L stimulation) within 15 minutes, compared with only a 40% reduction of HPR in the cangrelor treated group, and a negligible effect in the oral ticagrelor groups (chewed 0%, integral 5% reduction). The safety and efficacy of routine tirofiban administration in a contemporary STEMI treatment algorithm warrant further clinical investigation.

## 7. Critical Appraisal of Current Antithrombotic Regimens 

**Fig. 3.**
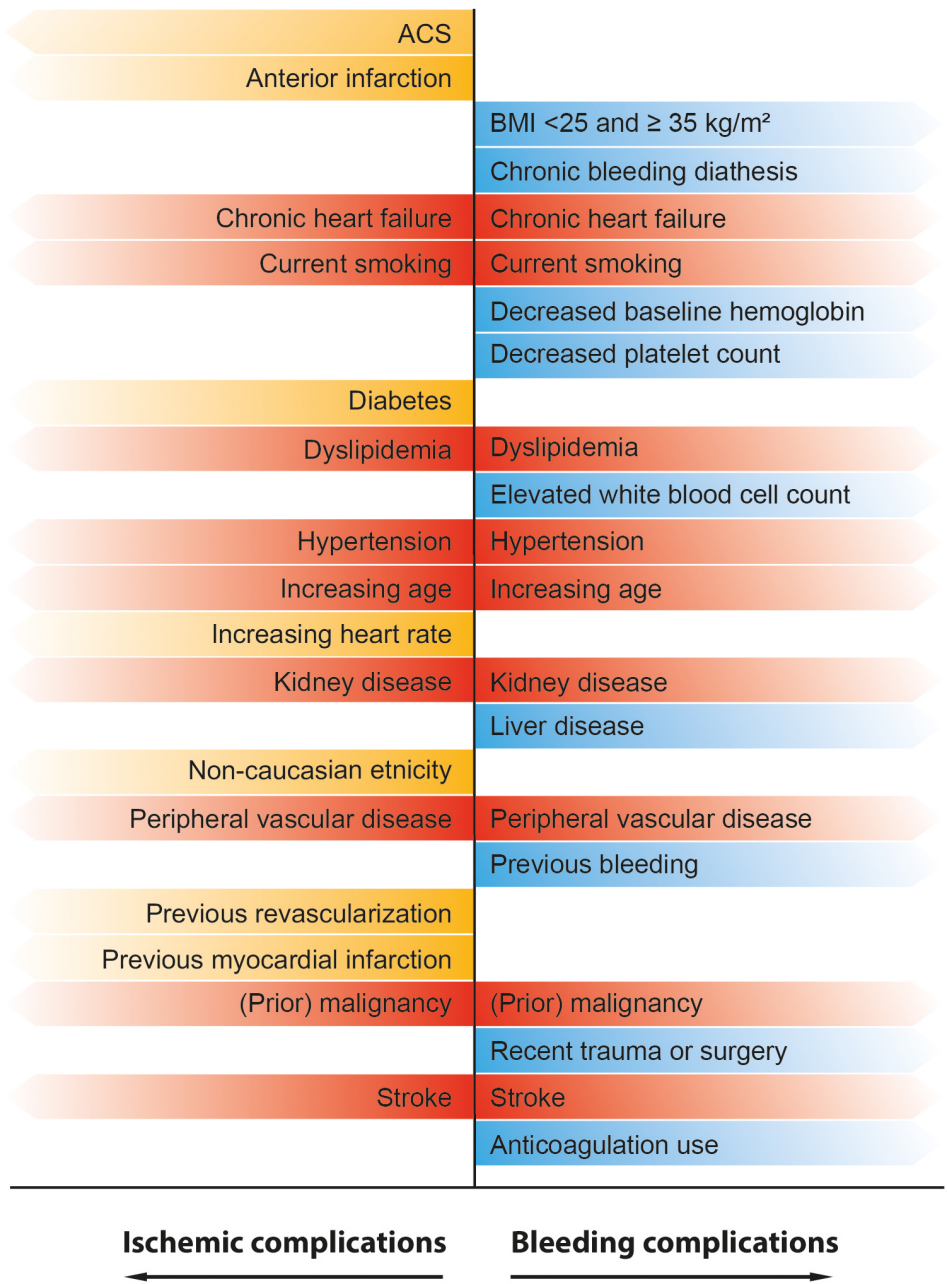
**Overview of previously identified predictors for ischemic and bleeding complications in patients with STEMI**. Yellow labeled predictors are associated with an increased risk of ischemic complications. Blue labeled predictors are associated with an increased risk of bleeding complications. Red labeled predictors represent a high-risk patient group that is susceptible for both bleeding and ischemic complications [171, 172, 173].

The development of antithrombotic therapy for has contributed to a reduction of ischemic complications and mortality in STEMI patients undergoing primary PCI. Importantly, the use of antithrombotic therapy must balance the decrease in ischemic complications with the risk of periprocedural bleeding. Unfortunately, this balance has proven to be challenging, since effective (combinations of) agents reducing ischemic complications often increase the rate of bleeding simultaneously [168]. Bleeding complications in patients with STEMI have an excess in death, recurrent MI, and stent thrombosis [28, 169, 170]. Interestingly, a large overlap exists in patient characteristics and risk factors for both ischemic and bleeding complications, underlining the complexity in the pursue of a patient-tailored antithrombotic approach. in which individual ischemic and bleeding risk should be weighed per patient (Fig. 3, Ref. [171, 172, 173]).

Aside from optimizing the contemporary treatment regimens, the search for novel antithrombotic agents with more favorable ischemic and safety profiles, is ongoing. Conceivably these agents will provide a solution for the limitations associated with currently available antithrombotic agents.

## 8. Future Directions in Antithrombotic Therapy

**Fig. 4.**
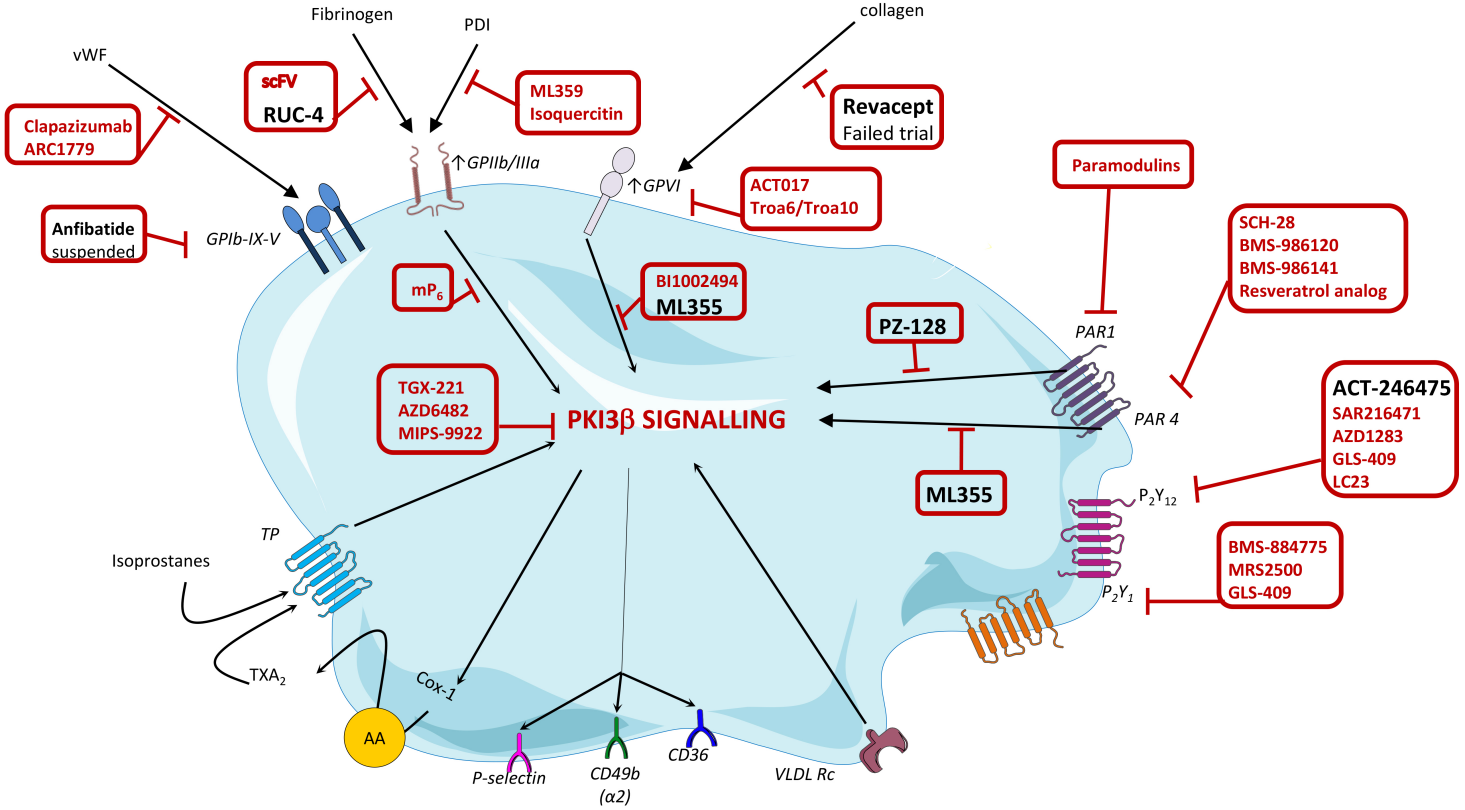
**Targets and novel platelet inhibitor molecules**. Depicted in red tentative new molecules in development against the indicated platelet receptors or signaling pathways. Depicted in black those novel molecules that have reached clinical development. *At GPIIb/IIIa level*: single-chain variable fragment (scFv) antibodies specifically target the high-affinity configuration ot the GPIIb/IIIa receptor; RUC-4 interferes with fibrinogen binding and the conformational change of the receptor from low- to the high-affinity state; ${\mathrm{mP}}_{6}$ is a myristoylated ExE motif peptide, which blocks the outside-in signaling of the GPIIb/IIIa receptor; Isoquercetin and ML359 are two agents inhibiting the protein disulfide isomerase (PDI) enzyme. *At GPVI level*: Revacept binds is a GPVI-collagen interaction inhibitor binding to exposed collagen at sites of plaque rupture; ACT017, ahumanized monoclonal antibody fragment has a high affinity for GPVI and a strong inhibitory efficacy; Troa6 and Troa10 peptides specifically antagonice the GPVI receptor. BI1002494 is Syk- inhibitor of downstream signaling. *At PAR level*: Paramodulins target the cytoplasmatic site of PAR 1, thereby inhibiting phosphatidylinositol 3 kinase b (PI3Kb) signaling; PZ-128 is a pepducin targeting the intracellular aspect of the PAR-1 receptor; BMS-986120 and BMS-986141 are specific inhibitors of the PAR-4 receptor; SCH-28 is a synthetic small molecular heparin analogue that inhibits PAR-4 by blocking thrombin exosite II. *At GPIb-IX-V level*: ARC1779 and caplacizumab bind to vWF- A1 domain; Anfibatide is a derivative of snake venom that blocks the interaction of GPIb and vWF (trial was suspended). *At ${\mathrm{P2Y}}_{12}$ and ${\mathrm{P2Y}}_{1}$ level*: BMS-884775 and MRS2500 are both ${\mathrm{P2Y}}_{1}$ receptor inhibitors; GLS-409 is a ${\mathrm{P2Y}}_{1}$ and ${\mathrm{P2Y}}_{12}$ inhibitor, while ACT-246475, SAR216471 and AZD1283 are selective ${\mathrm{P2Y}}_{12}$ inhibitors. *At signaling level*: TGX-221, AZD-6482, and MIPS-9922 are specific inhibitors of the PI3Kb signaling pathway; ML355 is a 12(S)-lipoxygenase (12-LOX) inhibitor that interferes with PAR-4, GPVI and FcgRIIa signaling pathways. *Modified from Badimon L, et al. (2014)* [174].

The search for new potent and safer antiplatelet agents has continued during the last years. Research has been focusing not only on blocking platelet surface receptors but also on interrupting/blocking different signaling pathways involved in platelet activation (Fig. 4, Ref [174]). Unfortunately, some of the new molecules have not reached yet the clinical arena due to difficulties in drug development or problems associated to unexpected bleeding or futility [175, 176].

Interestingly, Revacept, a novel GPVI antagonist was recently tested in the ISAR-PLASTER Phase 2 trial (ClinicalTrials.gov Identifier: NCT03312855) and failed to show any benefit added to standard therapy in the treated PCI patients [177].

Among those that have been advanced into clinical phase of development, as shown below, we can find one ${\mathrm{P2Y}}_{12}$ inhibitor (ACT-246475 or selatogrel), one inhibitor of GpIIB/IIIa (RUC-4) and one inhibitor of PAR1 signaling (PZ-128).

In a second tier with experimental data in humans and novel mechanism of action but not yet in clinical development we have ML355, that blocks Akt, PI3K and Erk1/2, but not p38, Syk or PLCγ2 phosphorylation in activated platelets. It is a selective 12-LOX inhibitor that blocks thrombosis with little effects on hemostasis [178, 179].

### 8.1 Selatogrel

Selatogrel is a potent, highly selective and reversible non-thienopyridine ${\mathrm{P2Y}}_{12}$ inhibitor [167]. Selatogrel is administered subcutaneously and provides a rapid dose-related antiplatelet effect within 15 minutes, which lasts for several hours [180, 181]. The pharmacodynamic effects of selatogrel is similar for both stable patients and patients presenting with ACS [180, 182]. Based on the pharmacokinetic and –dynamic profile, selatogrel appears to be a promising agent for the acute management of STEMI.

There is currently no phase III trials data available assessing the efficacy of selatogrel in patients with AMI. However, the randomized, multi-center, placebo-controlled SOS-AMI trial (ClinicalTrials.gov Identifier: NCT04957719) started patient recruitment in 2021 and is investigating the efficacy of self-administered selatogrel injections in patients at risk of recurrent AMI with new onset symptoms suggestive of AMI. This trial has the potential to contribute important data for the use of early prehospital antithrombotic therapies to prevent myocardial damage.

### 8.2 RUC-4

RUC-4 is a reversible αIIbβ3 inhibitor that provides a dose-dependent, rapid and near-complete platelet inhibition within 15 minutes after subcutaneous administration [183, 184].

In a phase II trial, the efficacy of RUC-4 is being assessed in 27 STEMI patients using escalating doses (0.075 mg/kg, 0.090 mg/kg, 0.011 mg/kg). A single subcutaneous injection of escalating doses of RUC-4 resulted in a dose-dependent, high-grade platelet inhibition effect within 15 minutes. Approximately one out of five patients experienced access-site hematomas, and two patients suffered from severe access-site related hematomas [185].

In 2021, the phase IIb, randomized, multi-center, placebo-controlled CELEBRATE trial (ClinicalTrials.gov identifier NCT04825743) started patient enrolment. This trial will determine the efficacy of RUC-4 as prehospital therapy in patients with STEMI.

### 8.3 Inhibitor of PAR1 Signaling (PZ-128)

PZ-128 is a first-in-class cell penetrating lipopeptide pepducin inhibiting the PAR-1-G protein signalling pathway. By targeting the intracellular surface of the receptor. It is a parenteral antiplatelet agent that seems to provide rapid, specific, dose-dependent, and reversible inhibition of platelet PAR-1 through a novel mechanism. The safety and efficacy of PZ-128 was first assessed in the TRIP-PCI (Thrombin Receptor Inhibitory Pepducin in Percutaneous Coronary Intervention) trial (ClinicalTrials.gov identifier NCT02561000) in patients undergoing cardiac catheterization with intent to perform percutaneous coronary intervention. In this first-in-patient setting, coadministration of PZ-128 with standard antiplatelet therapy appeared to be safe, well tolerated, and potentially reduced periprocedural myonecrosis. These results have provided a basis for further clinical trials [186].

## 9. Conclusions

Antithrombotic agents play a pivotal role in the procedural safety of STEMI patients undergoing primary PCI, and the prevention of peri-procedural thrombotic and post-procedural ischemic complications. Early initiated antiplatelet therapy, consisting of aspirin and a potent ${\mathrm{P2Y}}_{12}$ inhibitor, and anticoagulation therapy with UFH currently represent the mainstay of STEMI treatment. Several other antithrombotic agents—each with their particular risk-benefit profile—including GPIs, intravenous ${\mathrm{P2Y}}_{12}$ inhibitors and direct thrombin inhibitors are completing the current arsenal of antithrombotic therapeutics. Despite all the progress, contemporary antithrombotic therapy regimen is characterized by two major drawbacks: the delayed onset of antiplatelet effect by oral agents, and the increased risk of bleeding complications. Former might contribute to an increase in ischemic complications and alleviation of myocardial salvage accomplished by primary PCI. The latter remains the Achilles’ heel of antithrombotic therapy. Identification of ‘high risk’ patients remains challenging, due to the large overlap in risk factors for both ischemic and bleeding complications. This underscores the importance of meticulous use and appropriate dosing of existing antithrombotic therapy to minimize both ischemic and hemorrhagic complications during primary PCI, while efforts have to be preserved for developing new agents with improved safety and efficacy profiles.
